# Assessing matrix quality by Raman spectroscopy helps predict fracture toughness of human cortical bone

**DOI:** 10.1038/s41598-019-43542-7

**Published:** 2019-05-10

**Authors:** Mustafa Unal, Sasidhar Uppuganti, Selin Timur, Anita Mahadevan-Jansen, Ozan Akkus, Jeffry S. Nyman

**Affiliations:** 1 Department of Orthopaedic Surgery & Rehabilitation, Vanderbilt University Medical Center, Nashville, TN 37232 USA; 2 Center for Bone Biology, Vanderbilt University Medical Center, Nashville, TN 37232 USA; 3 Vanderbilt Biophotonics Center, Vanderbilt University, Nashville, TN 37212 USA; 4 Department of Biomedical Engineering, Vanderbilt University, Nashville, TN 37212 USA; 5 Department of Mechanical and Aerospace Engineering, Case Western Reserve University, Cleveland, OH 44106 USA; 6 Department of Orthopaedics, Case Western Reserve University, Cleveland, OH 44106 USA; 7 Department of Biomedical Engineering, Case Western Reserve University, Cleveland, OH 44106 USA

**Keywords:** Tissues, Translational research

## Abstract

Developing clinical tools that assess bone matrix quality could improve the assessment of a person’s fracture risk. To determine whether Raman spectroscopy (RS) has such potential, we acquired Raman spectra from human cortical bone using microscope- and fiber optic probe-based Raman systems and tested whether correlations between RS and fracture toughness properties were statistically significant. Calculated directly from intensities at wavenumbers identified by second derivative analysis, Amide I sub-peak ratio I_1670_/I_1640_, not I_1670_/I_1690_, was negatively correlated with K_init_ (N = 58; R^2^ = 32.4%) and J-integral (R^2^ = 47.4%) when assessed by Raman micro-spectroscopy. Area ratios (A_1670_/A_1690_) determined from sub-band fitting did not correlate with fracture toughness. There were fewer correlations between RS and fracture toughness when spectra were acquired by probe RS. Nonetheless, the I_1670_/I_1640_ sub-peak ratio again negatively correlated with K_init_ (N = 56; R^2^ = 25.6%) and J-integral (R^2^ = 39.0%). In best-fit general linear models, I_1670_/I_1640,_ age, and volumetric bone mineral density explained 50.2% (microscope) and 49.4% (probe) of the variance in K_init_. I_1670_/I_1640_ and v_1_PO_4_/Amide I (microscope) or just I_1670_/I_1640_ (probe) were negative predictors of J-integral (adjusted-R^2^ = 54.9% or 37.9%, respectively). While Raman-derived matrix properties appear useful to the assessment of fracture resistance of bone, the acquisition strategy to resolve the Amide I band needs to be identified.

## Introduction

The increase in fracture risk with aging is disproportionate to the decrease in bone mass that occurs after ~50 years of age in both women and men. A comprehensive, robust prediction of fracture risk, based on multiple aspects of bone, could improve the identification of those requiring an intervention, thereby reducing the economic burden and poor quality of life that fractures impose. Numerous changes in bone at multiple length-scales contribute to age- and disease-related decreases in the overall resistance of bone to fracture^1^. For example, lower mechanical competence of bone at the apparent level (independent of macro-structure but not micro- or ultrastructure) have been associated with an increase in cortical porosity (micro-scale)^2^, an increase in glycation-mediated, non-enzymatic collagen crosslinks (molecular-scale)^3^, an increase in degree of mineralization (micro-scale)^4^, an increase in mineral crystal size (nano-scale)^5^, an increase in collagen denaturation (nano-scale)^6^, and a decrease in matrix-bound water (molecular-scale)^7^; whereas, bending strength of the femoral neck and compressive strength of lumbar vertebra at the whole-bone level have been associated with decreases in bone mass^8^, thickness of the cortices^9^, volumetric bone mineral density (BMD)^10^, and trabecular thickness^11^.

Dual-energy X-ray absorptiometry (DXA) measures a subject’s areal BMD and bone mineral content at multiple sites, whereas current high-resolution peripheral quantitative computed tomography (HR-pQCT) and micro-magnetic resonance imaging (μMRI) provide clinical measurements of cortical micro-structure and trabecular architecture at peripheral sites. Such measurements including volumetric BMD from HR-pQCT are important contributors to bone fracture risk^12^. None of these clinical measurements can accurately distinguish individuals with osteoporosis from those without osteoporosis^13^, which is one reason why guidelines for treating a patient for osteoporosis are based on risk factors, not solely on imaging measurements of bone^14^. One barrier to establishing a robust, patient-specific predictor of fracture risk is the lack of a diagnostic tool that is sensitive to the contribution of the bone matrix to the fracture resistance of bone^1^. To date, there are no FDA-approved techniques for *in vivo* assessment of bone matrix quality.

The current available methods to assess characteristics of the bone matrix composition *ex vivo* include: (i) vibrational spectroscopy techniques such as Fourier Transform Infrared (FTIR) and Raman spectroscopy (RS), (ii) microscopy techniques such as scanning acoustic microscopy (SAM), atomic force microscopy (AFM), and quantitative backscatter and transmission electron microscopy imaging (qBEI and TEM), and (iii) wide-angle and small-angle X-ray diffraction/scattering techniques (WAXS and SAXS). While all these techniques provide useful information about the bone matrix^15^, RS is the only method that is both sensitive to composition and organization of all three primary components of bone (mineral, organic matrix, and water)^16–18^ and has clinical feasibility in the near term^19^.

Of the emerging techniques to assess the quality of a patient’s bone matrix, the OsteoProbe measures the resistance of periosteal bone to impact micro-indentation (bone material strength index or BMSi)^20^ whereas ultra-short echo-time (UTE) MRI measures the concentrations of matrix-bound water (C_bw_) and pore water (an indicator of porosity)^21^. The key determinants of BMSi and C_bw_ have yet to be identified. Therefore, RS is particularly well suited to help fulfill the unmet need of clinically assessing the quality of the bone matrix and to help identify mechanisms that increases fracture risk (beyond mineral density and bone structure).

RS measurements of the bone matrix calculated from either integrated area or peak intensity include: mineral-to-matrix ratio (ν_1_PO_4_/Amide I, ν_1_PO_4_/Proline and ν_2_PO_4_/Amide III), Type-B carbonate substitution (CO_3_/ν_1_PO_4_), crystallinity (the inverse of the line-width of the ν_1_PO_4_ peak at half the height from baseline or half-maximum; 1/FWHM)^22^, collagen crosslink or matrix maturity ratio (Amide I sub-band ratio at ~1670 cm^−1^ and ~1690 cm^−1^)^23^, and helical status of collagen (Amide I sub-peak ratio at ~1670 cm^−1^ and ~1640 cm^−1^)^24^. Using standard laboratory RS instruments (integrated into a compound microscope), RS measures were previously observed to significantly change with disease^22,25^ and drug therapy^16^ as well as to correlate with selected mechanical properties of bovine cortical bone^24,26,27^, rat cortical bone (aging study)^22^, and mouse cortical bone (genetic knock-out study)^23^. To the best of our knowledge, there are only two studies showing Raman measures can predict mechanical properties of human bone at the apparent-level^5,28^. In the study published by Yerramshetty and Akkus^5^, crystallinity explained 15.8% of the variance in elastic modulus and 7.1% of the variance in yield stress when RS analysis of tensile specimens were pooled from different quadrants of the femur mid-shaft. In the fracture toughness study involving specimens from one quadrant of the femur mid-shaft^24^, ν_1_PO_4_/Amide I ratio explained 10.9% the variance in J-integral and 11.6% of the variance in crack initiation toughness, whereas Type-B carbonate substitution explained 7.7% of the variance in crack growth toughness. Acquisition and data processing methods could potentially affect these correlations.

**Figure 1:**
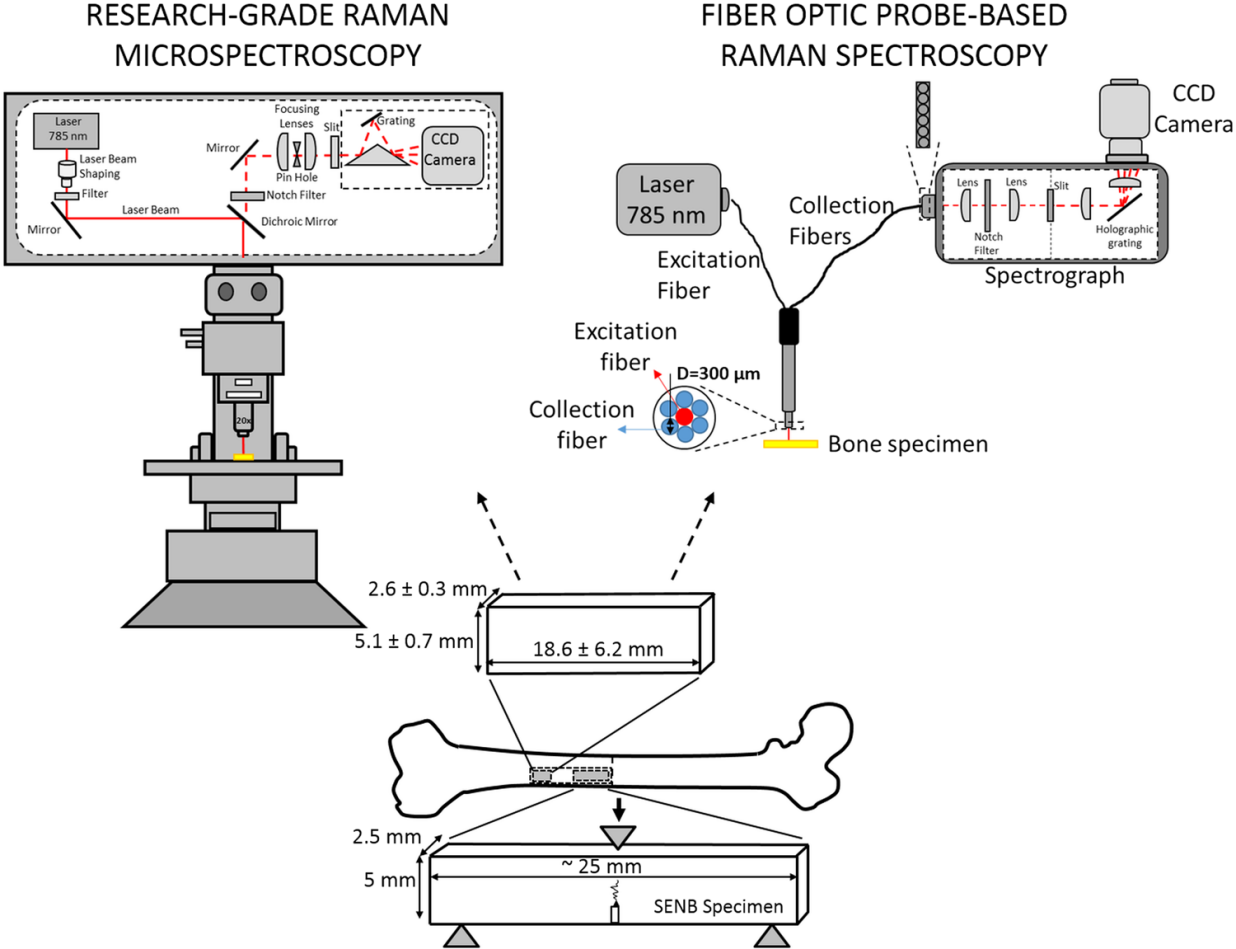
Schematic depiction of a research-grade, Raman micro-spectroscopy instrument and a fiber optic probe-based Raman spectroscopy instrument. Raman micro-spectroscopy involves mirrors, optical filters, focusing lenses, and objective lens to deliver the laser onto the specimen and direct the collection of Raman photons to the grating and CCD camera (**A**). Confocality is provided by a pin hole aperture. This type of RS preserves inherent laser polarization bias. Fiber optic probe-based Raman spectroscopy (Fiber optic RS) involves one fiber (300 μm in diameter) to deliver the laser onto the specimen and six collection fibers (each 300 μm in diameter) to direct collecting Raman photons onto a spectrograph coupled with a CCD camera. Spectrograph consists of several lenses, filters, and grating to split the laser into different wavelengths and deliver to CCD camera (**B**). Fiber optic RS does not preserve inherent polarization bias. A bone sample was extracted from the lateral quadrant of the femoral mid-shaft and machined into either a single-edge notched-beam specimen for fracture toughness testing (proximal end) or un-notched specimen for Raman analysis (distal end). Raman data collection was done using both research-grade RS and fiber optic probe-based RS from both long surfaces of each specimen.

Using the emerging method of spatially offset RS (SORS)^19,29–33^ and Raman tomography^34^, several groups have acquired Raman spectra from bone through soft tissue *in vivo*, showing the feasibility of using RS to assess the bone matrix of patients^19,35^. However, a significant challenge to the translation of RS into the clinic is a meaningful analysis of the spectra that provides measurements differentiating strong, tough bone from fragile, brittle bone. There is currently no consensus on which RS measures are reliable predictors of the fracture resistance of human bone. Moreover, there are few guidelines on which methods for calculating RS measures improve sensitivity to differences in mechanical properties of bone. Lastly, to date, there is no evidence showing that Raman analysis predicts mechanical properties of human bone when acquiring the spectra with a fiber-optic Raman probe that essentially involves a different optical set-up compared to research-grade Raman micro-spectroscopy instrument (Fig. 1).

To advance RS as a laboratory tool and to motivate the development of clinical Raman probes for native bone, we hypothesized that (i) both fiber-optic RS and commercial Raman micro-spectroscopy can both predict fracture toughness of human cortical bone through the direct analysis of Amide I sub-peak ratios (i.e., without band fitting), (ii) fiber optic RS properties of the Amide I band are comparable with Raman micro-spectroscopy properties, and (iii) RS does not simply provide a surrogate of BMD and provides unique predictors of fracture toughness properties.

## Results

### Correlations between Raman properties and fracture toughness properties

**Figure 2:**
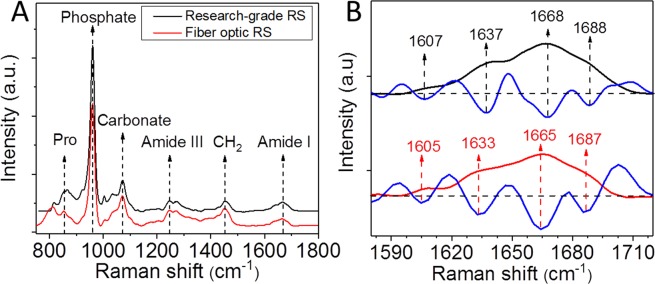
Raman spectra of bone collected by Raman micro-spectroscopy and by fiber optic probe-based RS. Overall both systems provide similar spectra with respect to wavelength location of peaks (**A**). The location of sub-peaks within the Amide I band were identified by local minima of the second derivative and thus slightly varied between research-grade RS and probe-based RS (**B**).

**Figure 3:**
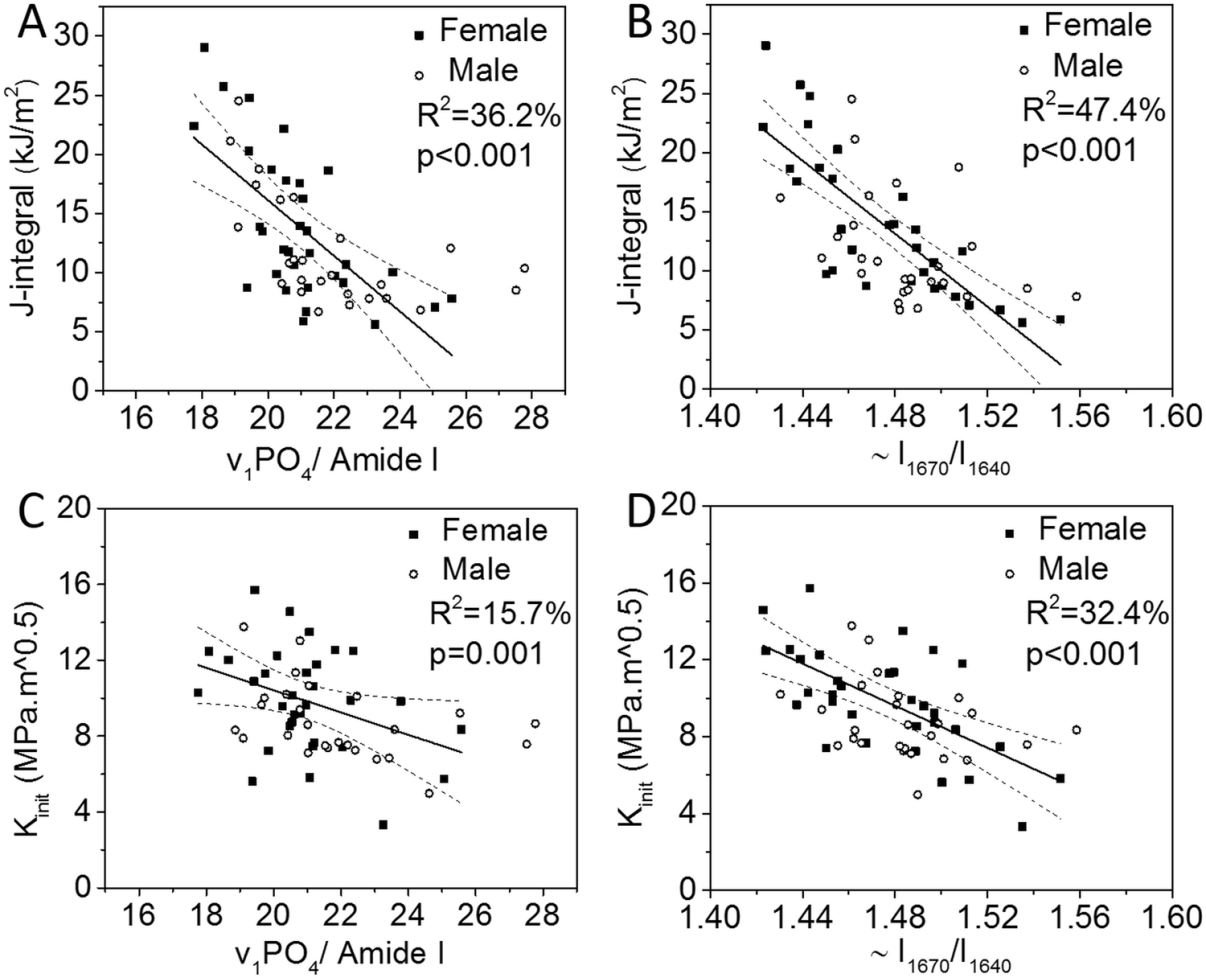
An Amide I sub-peak ratio and mineral-to-matrix ratios obtained by research-grade RS as potential predictors of fracture toughness. ν_1_PO_4_/Amide I (**A**) and ~I_1670_/I_1640_ (**B**) had the highest R^2^ values among the selected RS properties correlating with J-int. ν_1_PO_4_/Amide I (**C**) and ~I_1670_/I_1640_ (**D**) had the highest R^2^ values among the selected RS properties correlating with K_init_.

Both microscope- and fiber optic-based RS produced well-resolved Raman spectrum of human cortical bone in terms of peak locations and shapes of the bands, but with some subtle differences in the spectra between the instruments (Fig. 2). When analyzing the spectra collected by Raman micro-spectroscopy, all RS properties were directly related to age (Table 1). The correlation between ν_1_PO_4_/Amide I and age was only significant when female donors were removed, and CO_3_/ν_1_PO_4_ on average was higher for male than female donors at a given age (Supp. Mater. Fig. 1). The mineral-to-matrix ratio (MMR) by ν_1_PO_4_/Proline and the matrix maturity ratio by direct calculation of the ~I_1670_/I_1690_ ratio did not correlate with the fracture toughness properties, while other traditional RS properties (e.g., ν_1_PO_4_/Amide I and CO_3_/ν_1_PO_4_) and another Amide I sub-peak ratio ~I_1670_/I_1610_ negatively correlated with all three fracture toughness properties, explaining 5.0% to 36.2% of the variance (Fig. 3A–D and Table 1). The Amide I sub-peak ratio ~I_1670_/I_1640_ alone explained 47.4% of the variance in J-int (Fig. 3B and Tables 1), 32.4% of the variance in K_init_ (Fig. 3D and Tables 1), and 17.6% of the variance in K_grow_ (Table 1). Interestingly, there were no significant correlations between sub-band area ratios and the fracture toughness properties (Table 1) when these ratios were determined using the Amide I sub-band fitting technique.

**Table 1:** Statistically significant correlations exist between research-grade RS and fracture toughness properties.

Characteristic Property		Fracture Toughness			
		Age (N = 58)	K_init_ (N = 58)	K_grow_ (N = 47)	J-int (N = 58)
Age	(years)	1	(**−**) **21**.**9**^a^ *(0.0001)*	(**−**) **17**.**4** *(0.011)*	(**−**) **11**.**7** *(0.002)*
Bone mineral density	vBMD (mgHA/cm^3^)	NS	(**+**) **21**.**6** (0.018)	NS	NS
Mineral-to-matrix ratio	ν_1_PO_4_/Amide I	NS^a^	(**−**) **15**.**7** *(0.001)*	(**−**) **11**.**1** *(0.001)*	(**−**) **36**.**2** *(*<*0.001)*
	ν_1_PO_4_/Amide III	(**+**) **10**.**0**^b^ (0.004)	(**−**) **15**.**2** *(0.001)*	(**−**) **8**.**3** *(0.021)*	(**−**) **11**.**8** *(0.005)*
	ν_1_PO_4_/Proline	(**+**) **15**.**0** (0.001)	NS	NS	NS
	ν_1_PO_4_/CH_2_-wag	(**+**) **10**.**4** *(0.001)*	(**−**) **7**.**4** *(0.025)*	(**−**) **5**.**0** *(0.031)*	(**−**) **26**.**6** *(*<*0.001)*
Carbonate substitution	CO_3_/ν_1_PO_4_	(**+**) **35**.**5**^b^ *(*<*0.001)*	(**−**) **16**.**0** *(0.002)*	(**−**) **9**.**1** *(0.034)*	(**−**) **8**.**1** *(0.033)*
Crystallinity	1/FWHM(ν_1_PO_4_) (cm)	(**+**) **16**.**5** *(0.001)*	NS	(**−**) **13**.**3** *(0.012)*	NS
Matrix Maturity	~I_1670_/I_1690_ (direct^c^)	(**+**) **8**.**0** *(0.036)*	NS	NS	NS
	~A_1670_/A_1690_ (fitting^d^)	NS	NS	NS	NS
Helical status	~I_1670_/I_1640_ (direct)	(**+**) **20**.**1** *(*<*0.001)*	(**−**) **32**.**4** *(*<*0.001)*	(**−**) **17**.**6** *(*<*0.001)*	(**−**) **47**.**4** *(*<*0.001)*
	~A_1670_/A_1640_ (fitting)	NS	NS	NS	NS
Helical status	~I_1670_/I_1610_ (direct)	(**+**) **37**.**2** *(*<*0.001)*	(**−**) **14**.**1** *(0.003)*	(**−**) **9**.**6** *(0.023)*	(**−**) **17**.**1** *(0.001)*
	~A_1670_/A_1610_ (fitting)	NS	NS	NS	NS

R^2^ (%) in bold and corresponding p-values below 0.05 in *italics* as calculated from bootstrapped data. Otherwise, correlation was not statistically significant (NS).

^a^Both age and sex were significant covariates. ^b^The interaction between age and sex was significant such that correlation was significant for only male donors. See Supplemental Materials for linear regressions separated by sex. ^c^Sub-peak intensity ratio was directly calculated from the peak locations identified by the local minima of second derivative spectra. ^d^Sub-band area ratio was calculated by fitting 4 bands of Gauss/Lorentzian functions (variable mixture) within 5 wavenumbers of the second derivative locations.

Further addressing the possibility that the method of calculating RS properties influences the ability to predict fracture toughness, we also determined RS properties from integrated area ratios with and without the secondary linear baselines. In general, peak area ratios correlated with peak intensity ratios (Supp. Mater. Fig. 2), and the correlations between peak intensity ratios and fracture toughness properties (Table 1) remained significant when the secondary linear baseline correction was not applied prior to calculation (Supp. Mater. Table 1). However, the correlation between area ratio for ν_1_PO_4_/Amide I and K_int_ (as well as J-int) was no longer significant without the secondary linear baseline. Several other area ratios, CO_3_/ν_1_PO_4_ and ν_1_PO_4_/CH_2_, did not correlate with J-int and K_init_, respectively, regardless of whether secondary linear baseline correction was used (Supp. Mater. Table 1).

When 9 of 16 randomly selected spectra from the polished longitudinal surface were analyzed, all R^2^ values were reduced as compared to when RS properties from 32 averaged spectra were correlated with fracture toughness properties (Supp. Mater. Table 2). These values did not appear to depend on whether the 9 or 32 spectra per donor were averaged before peak ratio calculations or whether 9 or 32 peak ratios from individual spectrum were averaged per donor (Supp. Mater. Table 2).

**Figure 4:**
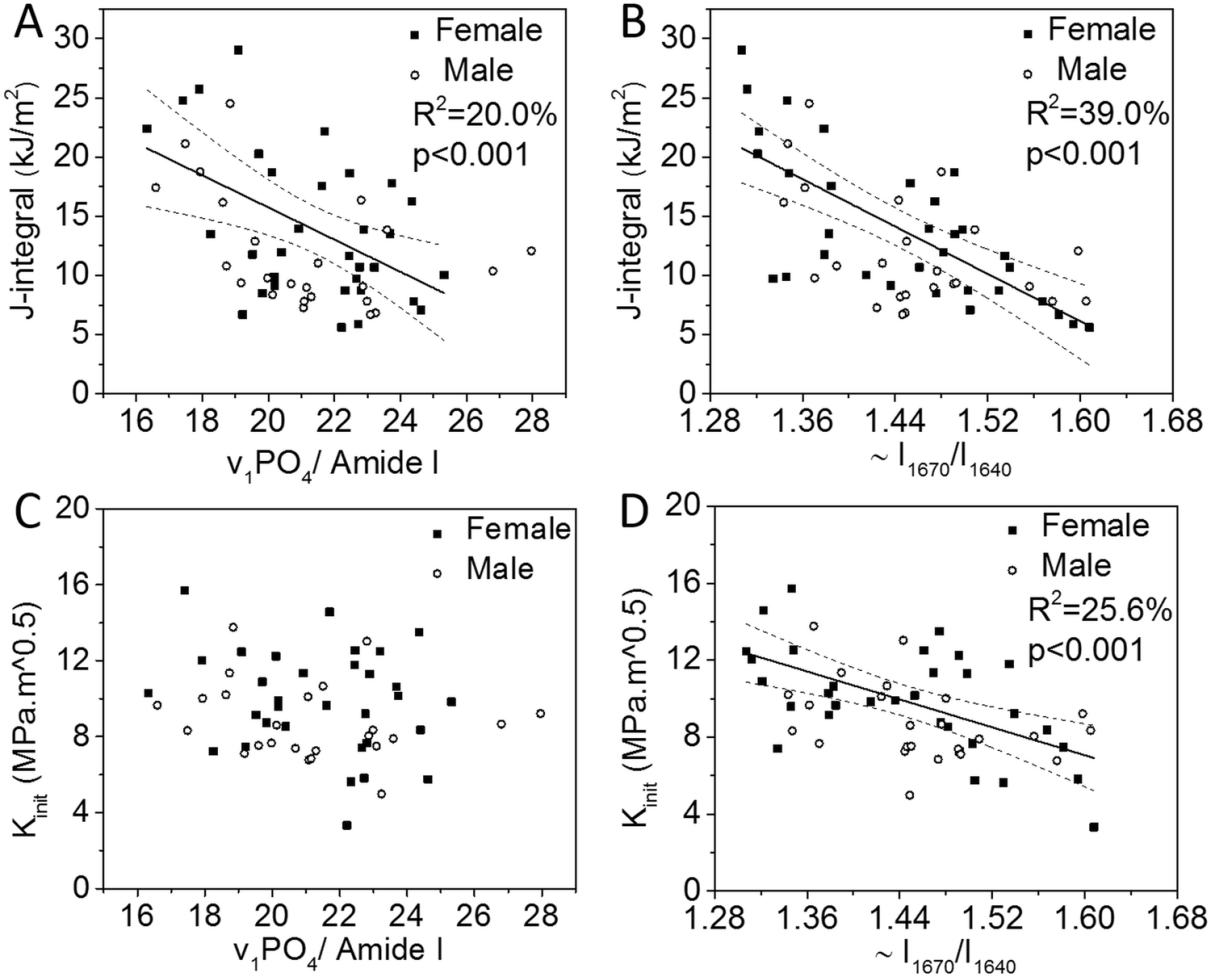
An Amide I sub-peak ratio and mineral-to-matrix ratio obtained by probe-based RS as potential predictors of fracture toughness. ν_1_PO_4_/Amide I (**A**) and ~I_1670_/I_1640_ (**B**) had the highest R^2^ values among the selected RS properties correlating with J-int. Although ν_1_PO_4_/Amide I correlated with K_init_ when obtained by research-grade RS, this ratio did not correlate with K_init_ when obtained by probe-based RS (**C**). ~I_1670_/I_1640_ had highest R^2^ value among the selected RS properties correlating with K_init_.

**Figure 5:**
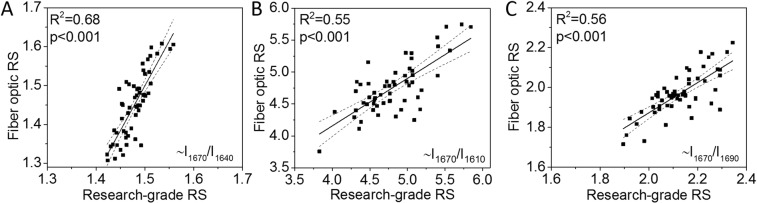
Amide I sub-peak ratios ~I_1670_/I_1640_ (A), ~I_1670_/I_1610_ (**B**), and ~I_1670_/I_1690_ (**C**) obtained by research-grade RS significantly correlated with the same ratios obtained by probe-based RS.

Similar to the results using the micro-spectroscopy system, Raman properties calculated from the spectra obtained by the fiber optic probe-based system had similar correlations between RS properties and age with CO_3_/ν_1_PO_4_ having the highest coefficient of determination (Table 2). There were however less significant correlations between probe-based RS properties and the fracture toughness properties (Table 2). The J-integral still correlated with ν_1_PO_4_/Amide I (Fig. 4A), but K_init_ no longer correlated with ν_1_PO_4_/Amide I (Fig. 4C). The Amide I sub-peak ratios ~I_1670_/I_1610_ and ~I_1670_/I_1640_, not the matrix maturity ratio, still negatively correlated with all three fracture toughness properties (Fig. 4B,D, and Table 2). The Amide I sub-peak ratios correlated across the two RS systems (Fig. 5A–C).

**Table 2:** Statistically significant correlations exist between fiber optic RS and fracture toughness properties.

Characteristic Property		Fracture Toughness			
		Age (N = 56)	K_init_ (N = 56)	K_grow_ (N = 45)	J-int (N = 56)
Age	(years)	1	(**−**) **22**.**7** *(0.0001)*	(**−**) **11**.**5** *(0.012)*	(**−**) **12**.**5** *(0.001)*
Bone mineral density	vBMD (mgHA/cm^3^)	NS	(**+**) **23**.**1** *(0.019)*	(**+**) **7**.**4** *(0.014)*	NS
Mineral-to-matrix ratio	ν_1_PO_4_/Amide I	NS	NS	NS	(**−**) **20**.**0** *(*<*0.001)*
	ν_1_PO_4_/Amide III	(**+**) **6**.**4**^a^ *(0.030)*	(**−**) **5**.**5** *(0.030)*	NS	NS
	ν_1_PO_4_/Proline	NS	NS	NS	NS
	ν_1_PO_4_/CH_2_	(**+**) **6**.**9** *(0.020)*	(**−**) **12**.**1** *(0.008)*	NS	(**−**) **15**.**6** *(0.005)*
Carbonate substitution	CO_3_/ν_1_PO_4_	(**+**) **34**.**3** *(*<*0.001)*	(**−**) **11**.**0** *(0.011)*	(**−**) **8**.**6** *(0.021)*	(**−**) **6**.**6** *(0.039)*
Crystallinity	1/FWHM(ν_1_PO_4_) (cm)	NS	NS	NS	NS
Matrix Maturity	~I_1670_/I_1690_ (direct^b^)	(**+**) **5**.**4** *(0.048)*	NS	NS	NS
	~A_1670_/A_1690_ (fitting^c^)	NS	NS	NS	NS
Helical status	~I_1670_/I_1640_ (direct)	(**+**) **12**.**1** *(0.002)*	(**−**) **25**.**6** *(*<*0.001)*	(**−**) **5**.**2** *(0.046)*	(**−**) **39**.**0** *(*<*0.001)*
	~A_1670_/A_1640_ (fitting)	NS	NS	NS	NS
Helical status	~I_1670_/I_1610_ (direct)	(**+**) **16**.**8** *(0.001)*	(**−**) **10**.**7** *(0.021)*	(**−**) **9**.**4** *(0.040)*	(**−**) **13**.**1** *(0.003)*
	~A_1670_/A_1610_ (fitting)	NS	NS	NS	NS

R^2^ (%) in bold a

nd corresponding p-values below (in *italics*) as calculated from bootstrapped data. Otherwise, correlation was not statistically significant (NS).

^a^The interaction between age and sex was significant. See Supplemental Materials for linear regressions separated by sex. ^b^Sub-peak intensity ratio was directly calculated from the peak locations identified by the local minima of second derivative spectra. ^c^Sub-band area ratio was calculated by fitting 4 bands of Gauss/Lorentzian functions (variable mixture) within 5 wavenumbers of the second derivative locations.

### Multivariate explanation of fracture toughness properties

To determine whether I_1670_/I_1640_ added value, it was included as covariate along with age and vBMD, two known determinants of fracture toughness, in general linear models (GLMs). Furthermore, other Raman properties with either the first or second highest R^2^ value (Table 1), were also included as covariates. The linear combination of vBMD and age together explained 40.7% of the variance in K_int_, whereas the linear combination of I_1670_/I_1640_ and age together only explained 35.2% of the variance (Table 3). Interestingly, when combining this Amide I sub-peak ratio with vBMD and age, all 3 variables were significant predictors (Table 3) explaining 50.2% of the variance in K_init_. The relative contributions to this variance were similar among 3 variables with age having the lowest standardized β coefficient (Table 3). vBMD did not become a significant explanatory variable of K_grow_ when it was included as a covariate of age; and although age correlated with K_grow_, it was no longer a significant explanatory variable when I_1670_/I_1640_ was included as covariate (Table 3). Combining age with crystallinity did not help explain the variance in K_grow_. With respect to the variance in J-int, age and vBMD together explained only 12.7% in which age had the higher β coefficient or stronger contribution (Table 3). When including I_1670_/I_1640_ as a covariate, age was no longer a significant predictor of J-int. Age and _V1_PO_4_/Amide I however were both significant explanatory variables in which the β coefficient of this mineral-to-matrix ratio was 2 times greater than that of age (Table 3). As such, the linear combination of ~I_1670_/I_1640_ and _V1_PO_4_/Amide I provided the best-fit model to predict J-int (Table 3).

**Table 3:** General linear models showing the combinations of properties (age, volumetric bone mineral density, and research-grade RS properties) that explain the variance in the fracture toughness properties of human cortical bone.

Fracture property	Explanatory variables	Linear models			Adj-R^2^ (%)
K_init_ (N = 58)	age	age (*β* = −0.47, <0.001)	—	—	**20**.**6**
	age + vBMD	age (*β* = −0.46, <0.001)	vBMD (*β* = 0.45, *p* < 0.001)	—	**40**.**7**
	age + RS	age (*β* = −0.27 *p* = 0.034)	—	~I_1670_/I_1640_ (*β* = −0.44, *p* < 0.001)	**35**.**2**
	age + vBMD + RS	age (*β* = −0.32, *p* = 0.006)	vBMD (*β* = 0.39, *p* = 0.001)	~I_1670_/I_1640_ (*β* = −0.36, *p* < 0.001)	**50**.**2**
K_grow_ (N = 47)	age	age (*β* = −0.42, *p* = 0.001)	—	—	**15**.**5**
	age + vBMD	age (*β* = −0.41, *p* = 0.004)	*vBMD* (*p* = 0.244)	—	**16**.**1**
	age + RS	*age* (*p* = 0.069)	—	~I_1670_/I_1640_ (*β* = −0.28, *p* = 0.017)	**20**.**2**
	age + RS	age (*β* = −0.32, *p* = 0.033)	—	*Crystallinity* (*p* = 0.074)	**18**.**4**
J-int (N = 58)	age	age (*β* = −0.34, *p* = 0.001)	—	—	**10**.**1**
	age + vBMD	age (*β* = −0.33, *p* = 0.008)	vBMD (*β* = 0.20, *p* = 0.050)	—	**12**.**7**
	age + RS	age (*β* = −0.26, *p* = 0.008)	—	ν_1_PO_4_/Amide I (*β* = −0.56 *p* < 0.001)	**40**.**7**
	age + RS	age (*p* = 0.805)	—	~I_1670_/I_1640_ (*β* = −0.66, *p* < 0.001)	**45**.**7**
	age + vBMD + RS	age (*β* = −0.26, *p* = 0.009)	*vBMD* (*p* = 0.105)	ν_1_PO_4_/Amide I (*β* = −0.54, *p* < 0.001)	**41**.**7**
	age + RS + RS	NA^a^	~I_1670_/I_1640_ (*β* = −0.47, *p* < 0.001)	ν_1_PO_4_/Amide I (*β* = −0.34, *p* = 0.002)	**53**.**9**

^a^Not applicable (NA) because age was not significant with the two Raman properties, which were significant covariates, and therefore not included in the best-fit model.

Similar to the Amide I sub-peak ratio from Raman micro-spectroscopy, the fiber optic probe-based I_1670_/I_1640_ was a significant covariate of age and vBMD in which the 3 variables explained 49.4% of the variance in K_init_ (Table 4). In this GLM, the contribution of age and I_1670_/I_1640_ to the variance was nearly equivalent (Table 3). The probe-based I_1670_/I_1640_ did not add value when included as covariate to age in predicting K_grow_ (Table 4). Although the probe-based ν_1_PO_4_/Amide I had a weak linear correlation with J-int (Table 2), it significantly explained the variance in J-int (adjusted-R^2^ = 33.1%) when combined with age and vBMD, which were also significant covariates. Also, ν_1_PO_4_/Amide I had the strongest contribution to the variance in J-int (i.e., highest β coefficient) compared to age and vBMD (Table 4) Nonetheless, probe-based ~I_1670_/I_1640_ without age, vBMD, or ν_1_PO_4_/Amide I provided the best explanation of the variance in J-int (Table 4).

**Table 4:** General linear models showing the combinations of properties (age, volumetric bone mineral density, and fiber optic RS properties) that explain the variance in the fracture toughness properties of human cortical bone.

Fracture property	Explanatory variables	Linear models			Adj-R^2^ (%)
K_init_ (N = 56)	age	age (*β* = −0.47, <0.001)	—	—	**21**.**2**
	age + vBMD	age (*β* = −0.44, <0.001)	vBMD (*β* = 0.44, *p* < 0.001)	—	**40**.**3**
	age + RS	age (*β* = −0.34, p = 0.005)	—	~I_1670_/I_1640_ (β = −0.38, p = 0.002)	**33**.**5**
	age + vBMD + RS)	age (*β* = −0.32, *p* = 0.002)	vBMD (*β* = 0.40, *p* < 0.001)	~I_1670_/I_1640_ (*β* = −0.33, *p* = 0.002)	**49**.**4**
K_grow_ (N = 45)	age	age (*β* = −0.34, p = 0.009)	—	—	**9**.**5**
	age + vBMD	age (*β* = −0.31, *p* = 0.034)	*vBMD* *(p* = *0.106)*	—	**13**.**1**
	age + RS	*age* *(p* = *0.066)*	—	*I*_*1670*_ */I*_*1640*_ *(p* = *0.479)*	**8**.**5**
	age + RS	*age* *(p* = *0.104)*	—	*I*_*1670*_ */I*_*1610*_ *(p* = *0.203)*	**10**.**9**
J-int (N = 56)	age	age (*β* = −0.35, *p* = 0.001)	—	—	**10**.**9**
	age + vBMD	age (*β* = −0.34, *p* = 0.008)	*vBMD* *(p* = *0.152)*	—	**12**.**7**
	age + RS	age (*β* = −0.37, *p* = 0.002)	—	ν_1_PO_4_/Amide I (*β* = −0.46, *p* < 0.001)	**31**.**2**
	age + RS	*age* *(p* = *0.169)*	—	~I_1670_/I_1640_ (*β* = −0.57, *p* = 0.001)	**38**.**9**
	age + vBMD + RS	age (*β* = −0.35, *p* = 0.002)	vBMD (*β* = 0.17, *p* = 0.035)	ν_1_PO_4_/Amide I (*β* = −0.45, *p* < 0.001)	**33**.**1**
	age + RS + RS	NA^a^	~I_1670_/I_1640_ (*β* = −0.53, *p* < 0.001)	*ν*_*1*_ *PO*_*4*_ */Amide I* *(p* = *0.098)*^*b*^	**40**.**0**

^a^Not applicable (NA) because age was not significant with the two Raman properties, which were significant covariates, and therefore not included in the model. ^b^Since ν_1_PO_4_/Amide I was not a significant explanatory variable without age as a covariate, the best-fit model includes ~I_1670_/I_1640_ (*β* = −0.63, *p* < 0.001) as the only predictor (adj-R^2^ = 37.9).

## Discussion

Matrix quality is an essential contributor to the overall fracture resistance of bone^1,36^. Unlike strength, which assesses the internal resistance of a material to irreversible deformation, fracture toughness is a measure of the ability of a material to resist crack growth. Given that a fracture is the culmination of cracks growing to catastrophic size, fracture toughness is a useful material property of bone to predict with non-destructive surrogates. Herein, we found that RS measurements obtained by a commercially available Raman micro-spectroscopy instrument correlated with several fracture toughness properties of human cortical bone. More importantly, several RS properties remained significantly correlated with the fracture toughness properties when the RS data were acquired using a fiber optic probe-based RS instrument. Also supporting the potential clinical utility of RS, a property related to collagen quality (~I_1670_/I_1640_) significantly explained the variance in both J-int and K_init._ It further improved the explanation of the variance in both J-int and K_init_ when known determinants of fracture toughness (age and vBMD) were included as covariates.

The resistance of bone to fracture involves numerous toughening mechanisms at different length scales across the hierarchical organization of the tissue^37^. These toughening mechanisms at the nanoscale include uncoiling of the triple helical collagen molecules and sliding of individual collagen molecules and mineralized collagen fibrils^37–40^. As with all materials, bone has pre-existing flaws from which cracks can initiate and grow (e.g., lacunae, fatigue-generated microcracks), and upon loading the ‘worst-case flaw’ (i.e., sharpened micro-notch in a single-edge notched beam or SENB specimen), the crack grows when the stress near notch tip reaches a critical value (K_init_). The resistance to further crack propagation, as reflected by K_grow_, depends on the ability of bone tissue to deflect the crack, thereby increasing the energy required to create fracture surfaces^37^. As such, additional toughening mechanisms at the submicron-to-micron level govern crack propagation including accumulation of diffuse damage in front of the crack tip^41^, mineralized collagen fibrils bridging cracks^42^, and crack deflection at cement lines^43^ or shifting lamellae with alternating collagen fibril direction^44^. We found that the ~I_1670_/I_1640_ ratio was better at explaining the variance in K_init_ than the variance in K_grow_, suggesting that crack initiation is more highly dependent on collagen integrity than crack growth. This is not a surprising outcome given that K_grow_ is also largely influenced by larger scale toughening mechanisms such as cement line density, cortical porosity, and tissue heterogeneity. Thus, finding a strong correlation between RS properties and K_grow_ may be rather difficult because RS properties are mostly associated with nanoscale toughening mechanisms. As such, a multifactorial assessment approach is likely necessary to accurately predict a patient’s fracture risk.

In previous studies, ~I_1670_/I_1640_ ratio increased upon thermally induced collagen denaturation and mechanically induced diffuse damage in bovine cortical bone^24^ as well as being higher in human cortical bone subjected to fatigue by rotating beam tests^45^. Unal *et al*.^24^ found that the ~I_1670_/I_1640_ ratio was negatively correlated with toughness and post-yield toughness of the bovine cortical bone obtained by three-point bending tests. ~I_1670_/I_1640_ ratio was thus proposed as a spectroscopic biomarker of the helical structure of collagen I, specifically indicative of a transition from a triple helical structure to less-ordered structure with perturbations in the molecular arrangement of α1 and α2 chains of collagen I^24^. Importantly, this Amide I sub-peak ratio in present study was also negatively correlated with fracture toughness properties, while the more widely reported matrix maturity ratio (~I_1670_/I_1690_) was not correlated with fracture toughness even when determined as a sub-band area ratio (~A_1670_/A_1690_). Raman sub-peaks of the Amide I band of bone are also sensitive to the disruption of collagen enzymatic crosslinking^46^, tissue aging^47^, degree of mineralization^48^, ionizing radiation^49,50^, and hydration^45^. More recently, the ~I_1670_/I_1640_ ratio was found to be higher in diabetic mouse bone with lower toughness compared to non-diabetic mice bone with higher toughness^51^, implying it could be sensitive to advanced glycated end-products (AGEs), although fluorescence AGEs and pentosidine, an AGE crosslink, were not different between the groups. The ~I_1670_/I_1640_ ratio was also recently reported to be sensitive to *in vitro* glycation of human cortical bone as well^52^. Cumulatively, all these findings indicate that several of the sub-peaks of Amide I are measures of the perturbation on the helical structure of collagen molecules, and perhaps reflect the capacity of collagen fibrils to dissipate energy, namely ~I_1670_/I_1640_ (Fig. 2B).

In our previous Raman micro-spectroscopy study using nearly the same donor set (N = 62, instead of N = 58) and acquisition of Raman spectra prior to fracture toughness testing^28^ in the region of crack propagation, we found either very weak positive correlations (ν_1_PO_4_/Amide I and CO_3_/ν_1_PO_4_) or no correlations between RS properties and fracture toughness properties; whereas in the present study, there were significant negative correlations for most of RS properties (Table 1). There are several differences in data collection process between the studies that potentially explain the discrepancy. In the previous study, we only collected 9 spectra at a 1 μm spot size within 0.25 mm^2^ area over the intended crack propagation region and calculated peak ratios for each Raman spectrum before averaging the RS properties per donor^28^; whereas in this study, we collected 32 spectra randomly distributed throughout the entire two longitudinal surfaces of neighboring bone specimens (~69 mm^2^ ± ~23 mm^2^) and determined the RS properties from averaged spectrum per donor (i.e., averaging the spectra minimizes noise). The number of spectra analyzed rather than averaging spectra before property calculation affected the coefficient of determination (Supp. Mater. Table 2). This is perhaps not surprising since bone composition is inherently heterogeneous due to osteonal remodeling. Acquiring spectra from many sites helps capture the overall bulk composition of each human sample. Furthermore, with Raman micro-spectroscopy, inherent laser polarization bias (i.e., sensitivity to collagen fibril orientation) is more prominent with higher numerical aperture (NA) of the objective^53^. In the previous study, spectra were acquired with 50 × (NA = 0.75) objective compared to 20 × (NA = 0.40) objective in the current study. While the orientation of the bone samples relative to the polarization axis of the laser was similar between studies (different research-grade Raman instruments though), differences in the sensitivity to fibril orientation may have caused differences in the ν_1_PO_4_/Amide I measurements between the studies. The negative correlations, albeit weak, between fracture toughness and MMR (Table 1) were unexpected, but perhaps a relatively low organic matrix (higher ν_1_PO_4_/Amide I) confers more brittle-like behavior.

Custom fiber optic probe-based RS have been developed for *in vivo* diagnostic applications, namely the detection of cancer^54^. Spatially offset Raman spectroscopy (SORS) is a variant of fiber optic RS that allows data collection from a target layer through a turbid sample, and first developed for collecting Raman spectra of bone through the skin^29^. SORS analysis of bone is an emerging technique in osteoporosis research^19^ and in detection of other bone diseases (i.e., osteogenesis imperfecta or OI)^35^. Thus far, two pilot studies showed the feasibility of *in vivo*, non-invasive clinical RS measurements to detect chemical compositional differences between osteoporotic and healthy subject with principal component analysis (PCA)^19^ and between OI and healthy subject with the analysis of v_1_PO_4_/Amide III ratio^35^. Neither of the studies analyzed sub-peak ratios of the Amide I band. As such, to date, a clinically viable tool that assesses collagen integrity has not been established, even though type I collagen has long been thought to be a primary determinant of toughness^55^ and fracture toughness^56^.

In the present study, a fiber optic probe-based RS that does not preserve the primary polarization of the laser (Fig. 1) was in direct contact with prepared bone samples and provided measurements that partially explain the variance in fracture toughness properties (Table 2), even when collecting five Raman spectra from one longitudinal surface (K_init_ and J-int vs. ~I_1670_/I_1640_ provided in Supp. Mater. Fig. 3). It remains to be seen whether further optimization of Raman probe can improve the ability of RS to predict the fracture toughness of cortical bone (e.g., collection of more Raman spectra per bone, different acquisition parameters and/or additional fiber optics to improve the signal-to-noise, use of polarization preserving fiber optics to capture orientation of the mineralized collagen fibrils, inclusion of a filter to select for water peaks that exist at higher wavenumbers, and using different spectrograph to improve spectral resolution).

This present work had several limitations. Because bone segments nearer to the site of crack propagation were used in other studies, we collected the RS data from distant segments of variable size (~25–35 mm distal from the micro-notch in the axial direction), instead of collecting Raman spectra prior to mechanical testing. These remaining segments from the original bone strip were subjected to more freeze-thaw cycles than the SENB specimens, and this may affect the strength of the correlations. Also, since the spatial and spectral resolution as well as the acquisition time including the number of acquired spectra was different between the two 2 RS instruments (research-grade RS had ~21% higher signal-to-noise ratio than did probe-based RS), there is a possibility that the number of significant correlations (Tables 1 and 2) could be equivalent between the two configurations (Fig. 1) once probe-based RS is optimized. The fiber optic probe also directly contacted the longitudinal surface of samples extracted the femur mid-shaft, and so, we do not know yet: (1) whether reported correlations will persist in spectra collected through skin and periosteum at a clinically accessible site such as the tibial mid-shaft and (2) whether bone matrix quality recorded at the mid-shaft will be predictive of fracture risk at relevant sites such as the femoral neck. Thus, additional work is necessary to determine whether a SORS technique or a minimally invasive technique (under local anesthesia) can adequately resolve the Amide I band using radiant exposures that do not damage tissues before the clinical assessment of collagen quality *in vivo* can be realized.

As discussed in our previous publication^28^, reported correlation strengths between fracture toughness properties and various bone properties are typically weak-to-moderate (r = ~0.5 or R^2^ = ~25%). Again, this is likely due to the numerous toughening mechanisms that bone possesses and part of the rationale behind explaining fracture toughness with several independent explanatory variables (Tables 3 and 4). While RS-derived properties, namely ~I_1670_/I_1640_ and ν_1_PO_4_/Amide I, improved upon age and vBMD in predicting fracture toughness (i.e., its inclusion either increased the adjusted coefficient of determination and/or superseded these other known determinants), nearly half the variance in K_init_ and J-int was not explained. This is perhaps not surprising for two reasons: (i) R-curve testing is inherently stochastic because the location of the micro-notch (which may or may not be near a pore or a cement line) and the features that propagating crack encounters (causing random deflections) cannot be controlled and (ii) other independent factors such as bound water^7^, porosity and osteonal area^57^, collagen network connectivity^58^, and small-scale heterogeneity in the matrix^59^ likely influence fracture toughness and are not necessarily related to RS-derived mineral-to-matrix ratio or marker of collagen I helical order.

In conclusion, Raman spectroscopy-derived matrix properties, namely the direct calculations of ~I_1670_/I_1640_ and v_1_PO_4_/Amide I, significantly correlated with the fracture toughness of human cortical bone, though correlations were not particularly strong being highest for the overall energy dissipated (R^2^ = 47% and 36%, respectively). Moreover, the significant correlations persisted when the spectra were acquired with fiber optic probe-based RS, and ~I_1670_/I_1640_ helped age and vBMD explain crack initiation toughness of human cortical bone. Development of probe-based RS instrument using either percutaneous or transcutaneous strategy could advance the clinical assessment of bone matrix quality.

## Materials and Methods

### Bone specimen preparation and study design

The Institutional Review Board at Vanderbilt University deemed that the use of cadaveric bone did not qualify as human subject research and so informed consent was not necessary. The sources of the femurs (Musculoskeletal Tissue Foundation, National Disease Research Interchange, and Vanderbilt Donor Program) did not provide personally identifiable information (i.e., tissue was de-identified). All methods for processing and analyzing the bone followed relevant guidelines and regulations. We described the preparation of the mechanical specimens and measurement methods in our previous study^7^, and so they are briefly summarized herein. The proximal end of ~70 mm strips of cortical bone from cadaveric femur mid-shafts (lateral quadrant) were machined into single-edge notched beam (SENB) specimens (length x thickness x width of ~20–30 mm × ~2.5 mm × ~5 mm). The crack path region was scanned by micro-computed tomography (µCT) at an isotropic voxel size of 5 µm, and the corresponding volumetric BMD (vBMD) was determined. The SENB specimens were subjected to three-point bending using a progressive, cyclic loading protocol (loaded to + 0.07 mm at 0.01 mm/s and then-unload to −0.04 mm at 0.015 mm/s) with a short dwell period prior to the next load cycle^7^. The span was ~20 mm (4 × width), and the crack propagate perpendicular to the osteonal direction^7^. A non-linear fracture mechanics approach (R-curve testing) was used to determine transverse fracture toughness properties, namely crack initiation (K_init_), crack growth toughness (K_grow_) and J-integral (J-int) or the overall energy dissipated to propagate the crack to failure^7^. K_grow_ could not be calculated for specimens that fractured in a brittle manner. Additional specimens distal to the SENB were also cut (Fig. 1) from the remaining strip of cortical bone. For the present study, the most distal segment was available for 58 (28 males and 30 females spanning 21 to 101 years of age) of the original 62 donors (mean ± SD): length of 18.6 ± 6.2, width of 5.1 ± 0.7, and 2.6 ± 0.3). Two distal samples from male donors (21 years and 91 years of age) were not available for the fiber optic probe-based RS measurements. Samples were soaked in phosphate buffered saline and stored at −20 °C when not being analyzed and thawed to room temperature prior to Raman analysis.

### Raman spectroscopy

In this study, we collected Raman spectra from bone specimens using two RS systems: (i) confocal Horiba RS (Xplora, Horiba Jobin Yvon, Edison, NJ) with a 785 nm diode laser and with a 1200 lines/mm grating providing ~1.25 cm^−1^ spectral resolution and (ii) portable fiber optic probe-based RS. The probe-based RS involved: (i) an imaging spectrograph (Holospec f/1.8i, Kaiser Optical Systems, Ann Arbor, MI) coupled to a thermoelectrically cooled CCD camera (PIXIS: 256BR, Princeton. Instruments, Princeton, NJ), providing ~3.50 cm^−1^ spectral resolution, (ii) a 785 nm diode laser (Innovative Photonic Solutions, Monmouth Junction, NJ), and (iii) a custom-made fiber optic probe (EmVision, Loxahatchee, FL) consisting of one excitation and six collection fibers (each 300 µm in diameter) configured as a ring shape (Fig. 1). Wavelength calibration of the portable probe-based RS system was done using a neon-argon lamp. Naphthalene and acetaminophen standards were also used to determine the exact excitation wavelength for subsequent Raman shift calculations. The spectral response of the system was further corrected using a tungsten lamp calibrated by the National Institute of Standards and Technology.

For Raman micro-spectroscopy, the long axis of each specimen was aligned parallel to the axis of the primary laser polarization, and thirty-two Raman spectra per specimen were each obtained as the average of 12 consecutive spectra per spot with a 5-second acquisition using a 20x objective (NA = 0.40). Laser power was ~35 mW. For fiber-optic RS with a larger laser spot size than a 20x objective (~300 µm vs. ~2.5 µm), ten spectra per sample were each obtained as the average of 10 consecutive spectra per spot with 3-second acquisition, and laser power was set up at ~80 mW. The long axis was not specifically aligned with the polarization axis of the laser because fiber optics scramble the orientation of the light (Supp. Mater. Fig. 4). Raman data collection were randomly distributed throughout the entire two longitudinal surfaces of bone specimens (sixteen Raman spectra and five Raman spectra per surface for research-grade RS and fiber optic RS, respectively). Since the bone specimens were not immersed in PBS during the acquisition of the multiple spectra, some dehydration occurred. To verify that this does not affect the spectra, we collected spectra from 6 bone specimens before and after 20 min in air which is the maximum time for total spectra collection in this study. We found that there were no apparent differences in the RS measures between these two time points (Supp. Mater. Fig. 5) indicating partial air-drying for 20 minutes did not affect significantly the RS properties.

### Raman data analysis

Raman spectra were processed using LabSpec 5 software (Horiba Jobin Yvon, Edison, NJ) and a custom MATLAB script. First, Raman raw spectra collected at thirty-two or ten sites per bone specimen were averaged. Then, background fluorescence was removed from all averaged spectra by subtracting a 5^th^-order polynomial function from the base of the raw spectra (Supp. Mater. Fig. 6A). Then, the averaged spectra without normalizing to a mean or selected peak intensity were further smoothed to minimize noise using a proprietary de-noising (D-n) algorithm provided by the LabSpec 5 software. Before calculating RS properties, a secondary 5-point linear baseline was applied to minimize residual fluorescence (Supp. Mater. Fig. 6B,C).

From the averaged and de-noised spectrum per donor, we calculated the following RS properties from peak intensities (Supp. Mater. Fig. 6B,C): mineral-to-matrix ratio (ν_1_PO_4_/Amide I, ν_1_PO_4_/Proline, ν_1_PO_4_/Amide III and ν_1_PO_4_/CH_2_-wag), Type-B carbonate substitution (CO_3_/ν_1_PO_4_), crystallinity (the inverse of the line-width of the ν_1_PO_4_ peak at half the height from baseline or half-maximum; 1/FWHM), collagen crosslinks/matrix maturity ratio (calculated as the intensity at ~1670 cm^−1^ per intensity at ~1690 cm^−1^ or I_1670_/I_1690_), and the newly developed I_1670_/I_1610_ and I_1670_/I_1640_ ratio associated with collagen conformational change^24^. The locations of Amide I shoulders were not fixed at set wavenumbers but rather identified directly from the local minima of each second derivative (Fig. 2B). We also fitted four sub-peaks within the Amide I band using Gaussian-Lorentzian mixed functions. Briefly, each sub-band with a Gauss/Lorentzian mixture of 50%/50% was first centered on the 4 wavenumber locations that were identified by the second derivative analysis. Then, a non-linear constrained optimization algorithm in MATLAB determined the final wavenumber location of each peak within a ± 5 cm^−1^ window as well as the final mixture for each sub-band that minimized the weighted root mean square error between the experimental data and the sum of the 4 sub-peaks (Supp. Mater. Fig. 7)^52^. Finally, the Amide I sub-band ratios (A_1670_/A_1610_, A_1670_/A_1640_, A_1670_/A_1690_) were the respective sub-band area ratios.

We further processed our raw data from research-grade, commercial Raman micro-spectroscopy using three additional methods: (1) only 5^th^ order polynomial fluorescence subtraction (i.e., without a 5-point linear baseline correction) and then calculating ratios based on the intensity of the peaks, (2) same 5^th^ order polynomial fluorescence subtraction and then calculating area ratios in which the integration range of each band was modified from those used in Hammond *et al*.^60^ to match our Raman spectral acquisitions (Supp. Mater. Fig. 6D), and (3) calculated the integrated area ratios (same range used for each peak) from our initial spectra (5^th^ order polynomial fluorescence subtraction with a 5-point linear baseline correction, Supp. Mater. Fig. 6B,C). Noise filtering did not vary among the four approaches. To determine whether the number of spectra analyzed affected correlations, 9 randomly chosen spectra out of the 16 acquired from the polished longitudinal surface were averaged prior to RS property calculation. Lastly, RS properties were determined from individual spectrum per donor and then averaged (9 or 32 spectra).

### Statistical analysis

All statistical analyses were performed using STATA 12 statistical software (StataCorp LP, College Station, TX, USA) in which bootstrapping (1000 replicates) was used to generate the p-values since the normality assumption in regression analysis did not hold for most properties. Linear correlations between the Raman properties or vBMD and fracture toughness properties or age were determined at a significance level of 0.05. As described in our previous studies^57^, analysis of covariance was used to determine whether sex was a significant covariate of age in the explanation of each bone property (Supp. Mater. Table 3 and Supp. Mater. Fig. 1). Next, age, vBMD, and Raman properties were considered as independent predictors in general linear models with the fracture toughness properties as the dependent variables. The interaction terms and then the independent variables were removed in a step-wise backwards manner to determine which combination of properties best explained the variance in fracture toughness properties (i.e., highest adjusted R^2^).

### Ethical approval

Upon review of the project, the IRB at the corresponding author’s institution deemed the use of cadaveric tissue as non-human subjects research. All donors were de-identified by the allograft banks or donor services.

## Supplementary information

Supplementary Materials

## Data Availability

All Raman spectroscopy data, mechanical data, and micro-computed tomography data that were used in the correlation analyses are available from the corresponding author upon request.
