# Supplementary material for: Assessing matrix quality by Raman spectroscopy helps predict fracture toughness of human cortical bone
Source: Sci Rep. 2019 May 10;9:7195. doi: 10.1038/s41598-019-43542-7 (PMC6510799; doi:10.1038/s41598-019-43542-7)
Supplement: Supplementary file 1 — Supplementary Materials [file 41598_2019_43542_MOESM1_ESM.docx]

**SUPPLEMENTAL MATERIALS**

**Assessing matrix quality by Raman spectroscopy helps predict fracture toughness of human cortical bone**

Mustafa Unal, Sasidhar Uppuganti, Selin Timur, Anita Mahadevan-Jansen, Ozan Akkus, and Jeffry S. Nyman

**
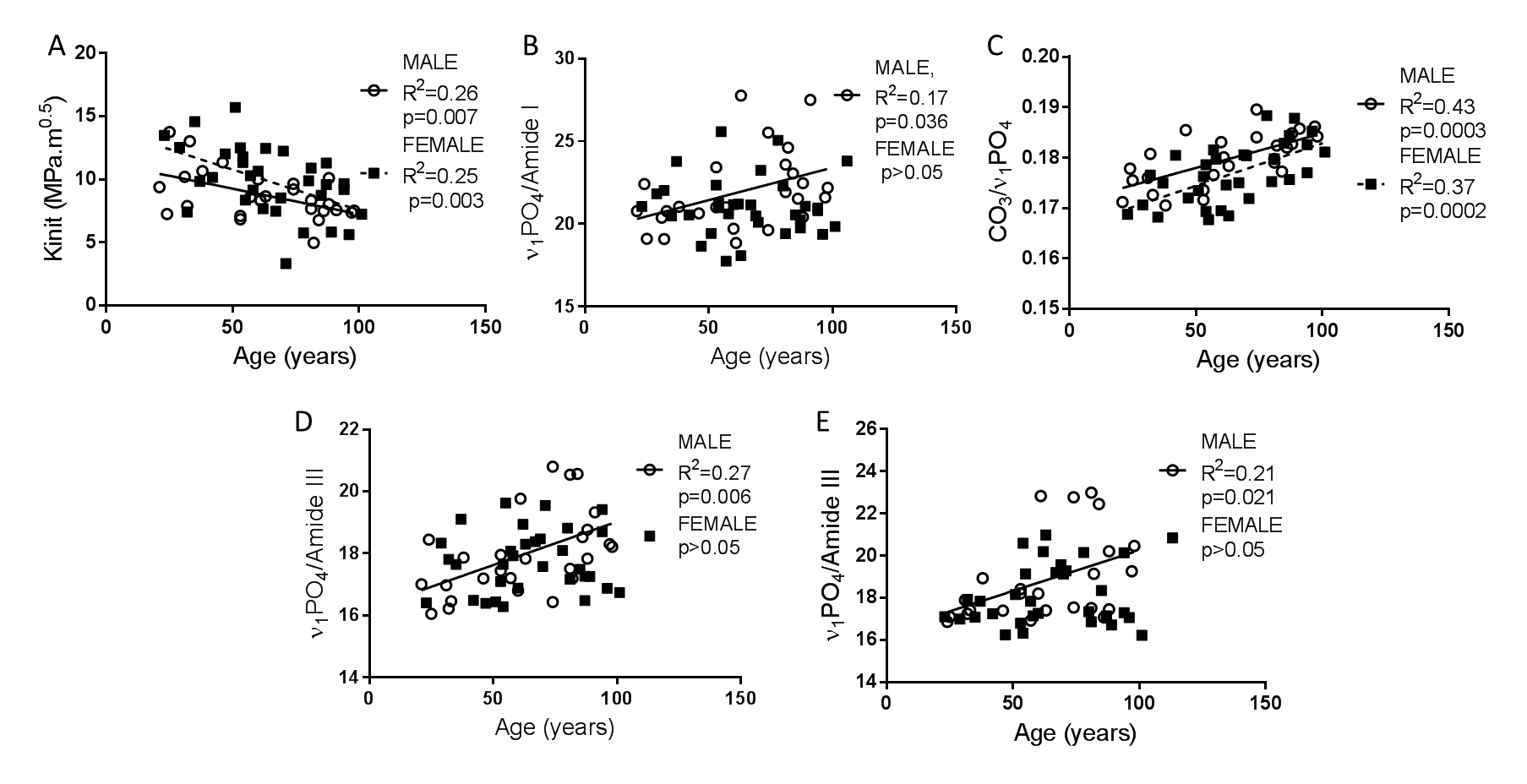
**

**Supplemental Figure 1:** Linear regressions between selected properties and age separated by sex. (A) K_init_: R^2^ = 0.25, p=0.003 for female and for R^2^ = 0.26, p=0.007 for male. (B) ν_1_PO_4_/Amide I obtained by Raman micro-spectroscopy: p=0.38 (NS) for female and R^2^ = 0.17, p=0.036 for male. (C) CO_3_/ ν_1_PO_4_ obtained by Raman micro-spectroscopy: R^2^ = 0.37, p<0.001 for female and for R^2^ = 0.43, p<0.001 for male. (D) ν_1_PO_4_/Amide III obtained by Raman micro-spectroscopy, p=0.65 (NS) for female and for R^2^ = 0.27, p=0.006 for male. (E) ν_1_PO_4_/Amide III obtained by fiber optic RS, p=0.72 (NS) for female and for R^2^ = 0.21, p=0.021 for male.


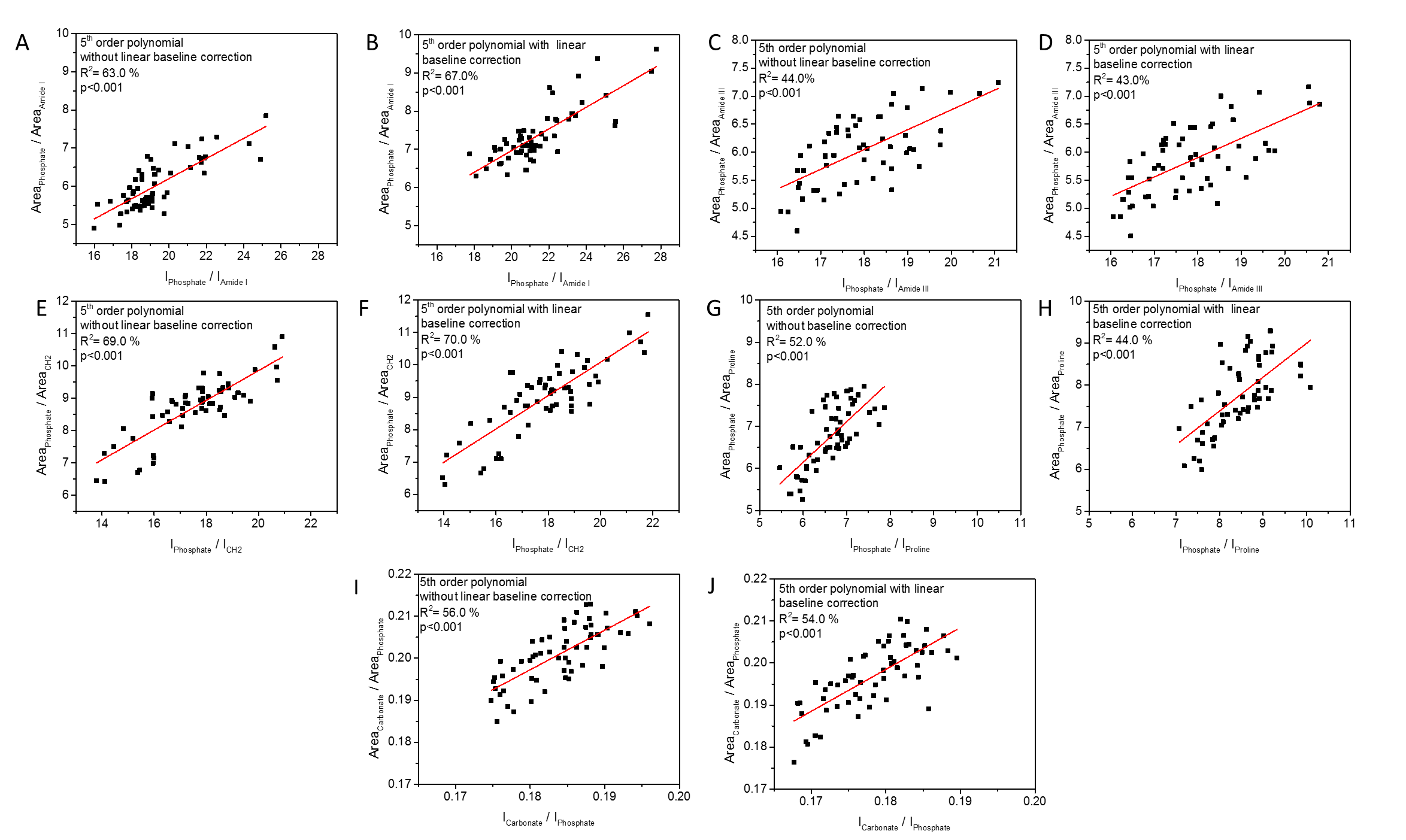


**Supplemental Figure 2:** There are moderate to strong correlations (R^2^ values range between 44.0 % to 70.0%) between Raman properties calculated from peak intensity (I) and those calculated from integrated area (Area) regardless of baseline correction approaches, which were 5^th^ order polynomial fluorescence subtractions with (B, D, F, H, J) and without linear baseline correction (A, C, E, G, I).

**Supplemental Table 1:** R^2^ values (p-values) for different spectral processing techniques to determine the Raman properties, namely intensity ratios and peak area ratios with and without the secondary linear baseline (all significant correlations were negative). NS: Not significant, N/A: Not applicable because sub-bands could not be properly fitted to the Amide I band without the secondary linear correction of the Amide I band. Amide sub-peak intensity ratios were directly calculated using the locations identified by the local minima of the second derivative spectrum.

| *Characteristic Property* | | *K_init_* | | | | *K_grow_* | | | | *J-integral* | | | |
| --- | --- | --- | --- | --- | --- | --- | --- | --- | --- | --- | --- | --- | --- |
|  |  | ***5^th^ order polynomial with linear baseline correction***  ***(Intensity ratios)*** | *5^th^ order polynomial without linear baseline correction*  *(Intensity ratios)* | *5^th^ order polynomial with linear baseline correction*  *(Area*  *Ratios)* | *5^th^ order polynomial without linear baseline correction*  *(Area ratios)* | ***5^th^ order polynomial with linear baseline correction***  ***(Intensity ratios)*** | *5^th^ order polynomial without linear baseline correction*  *(Intensity ratios)* | *5^th^ order polynomial with linear baseline correction*  *(Area*  *Ratios)* | *5^th^ order polynomial without linear baseline correction*  *(Area ratios)* | ***5^th^ order polynomial with linear baseline correction***  ***(Intensity ratios)*** | *5^th^ order polynomial without linear baseline correction*  *(Intensity ratios)* | *5^th^ order polynomial with linear baseline correction*  *(Area*  *Ratios)* | *5^th^ order polynomial without linear baseline correction*  *(Area ratios)* |
| Mineral-to-matrix ratio | ν_1_PO_4_/Amide I | **(-) 15.7** *(0.001)* | **(-) 8.9**  (0.023) | **(-) 16.9**  *(0.001)* | NS | **(-) 11.1**  *(0.001)* | **(-) 10.0**  *(0.030)* | **(-) 16.3**  *(0.000)* | **(-) 11.0**  *(0.007)* | **(-) 36.2**  *(<0.001)* | **(-) 20.5**  *(<0.001)* | **(-) 19.5**  *(0.001)* | NS |
|  | ν_1_PO_4_/Amide III | **(-) 15.2**  *(0.001)* | **(-) 15.5**  *(0.002)* | **(-) 6.1**  (0.023) | **(-) 6.9**  *(0.046)* | **(-) 8.3**  *(0.021)* | **(-) 7.5**  *(0.027)* | **(-) 10.4**  (0.008) | **(-) 10.1**  *(0.015)* | **(-) 11.8**  *(0.005)* | **(-) 11.0**  *(0.011)* | **(-) 10.9**  (0.012) | **(-)9.6**  *(0.019)* |
|  | ν_1_PO_4_/Proline | NS | NS | NS | NS | NS | NS | NS | NS | NS | NS | NS | NS |
|  | ν_1_PO_4_/CH_2_ | **(-) 7.4**  *(0.025)* | **(-) 7.2**  *(0.041)* | NS | NS | **(-) 5.0**  *(0.031)* | **(-) 5.1**  *(0.041)* | **(-) 5.6**  *(0.014)* | **(-) 7.2**  *(0.003)* | **(-) 26.6**  *(<0.001)* | **(-) 25.9**  *(<0.001)* | **(-) 24.3**  *(<0.001)* | **(-) 25.2**  *(<0.001)* |
| Carbonate  substitution | CO_3_/ν_1_PO_4_ | **(-) 16.0**  *(0.002)* | **(-) 21.4**  *(0.001)* | **(-) 8.1**  *(0.031)* | **(-) 11.8**  *(0.008)* | **(-) 9.1**  *(0.034)* | **(-) 10.3**  *(0.020)* | NS | **(-) 6.8**  0.045 | **(-) 8.1**  *(0.033)* | **(-) 13.5**  *(0.005)* | NS | NS |
| Crystallinity | 1/FWHM  (ν_1_PO_4_) | NS | NS | NS | NS | **(-) 13.3**  *(0.012)* | **(-) 11.1**  *(0.022)* | **(-) 13.3**  *(0.012)* | **(-) 11.1**  *(0.022)* | NS | NS | NS | NS |
| Matrix Maturity | ~I_1670_/I_1690_ | NS | NS | N/A | N/A | NS | NS | N/A | N/A | NS | NS | N/A | N/A |
| Helical Status | ~I_1670_/I_1640_ | **(-) 32.4**  *(<0.001)* | **(-) 28.9**  *(<0.001)* | N/A | N/A | **(-) 17.6**  *(<0.001)* | **(-) 14.4**  *(0.001)* | N/A | N/A | **(-) 47.4**  *(<0.001)* | **(-) 44.9**  *(<0.001)* | N/A | N/A |
| Helical Status | ~I_1670_/I_1610_ | **(-) 14.1**  *(0.003)* | NS | N/A | N/A | **(-) 9.6**  *(0.023)* | **(-) 6.6**  *(0.045)* | N/A | N/A | **(-) 17.1**  *(0.001)* | **(-) 8.1**  *(0.029)* | N/A | N/A |

**Supplemental Table 2:** R^2^ values (p-values) for Raman properties determined from averaged Raman spectrum per donor and determined from each individual spectra and then averaged both calculated for 32 and 9 sites of data collection points (all significant correlations were negative). NS: Not significant. * Raman properties were calculated from averaged Raman spectra per bone specimen ** Raman properties were calculated from individual Raman spectrum, and then averaged the final Raman properties values.

| *Characteristic Property* | | *K_init_* | | | | *K_grow_* | | | | *J-int* | | | |
| --- | --- | --- | --- | --- | --- | --- | --- | --- | --- | --- | --- | --- | --- |
|  |  | ***Averaged Raman spectra****  ***(32 spectra)*** | ***Individual Raman spectrum** (32***  ***spectra)*** | ***Averaged Raman spectra***  ***(9 randomly selected spectra)*** | ***Individual Raman spectrum***  ***(9 randomly selected spectra)*** | ***Averaged Raman spectra***  ***(32 spectra)*** | ***Individual Raman spectrum (32 spectra)*** | ***Averaged Raman spectra***  ***(9 randomly selected spectra)*** | ***Individual Raman spectrum***  ***(9 randomly selected spectra)*** | ***Averaged Raman spectra***  ***(32 spectra)*** | ***Individual Raman spectrum (32 spectra)*** | ***Averaged Raman spectra***  ***(9 randomly selected spectra)*** | ***Individual Raman spectrum (9 randomly selected spectra)*** |
| Mineral-to-matrix ratio | ν_1_PO_4_/Amide I | **(-) 15.7** *(0.001)* | **(-) 14.8**  *(0.001)* | **(-) 9.1**  *(0.022)* | **(-) 8.9**  *(0.041)* | **(-) 11.1**  *(0.001)* | **(-) 11.0**  *(0.003)* | **(-) 8.1**  *(0.026)* | **(-) 7.0**  *(0.036)* | **(-) 36.2**  *(<0.001)* | **(-) 33.4**  *(<0.001)* | **(-) 20.9**  *(0.002)* | **(-) 18.1**  *(0.003)* |
|  | ν_1_PO_4_/Amide III | **(-) 15.2**  *(0.001)* | **(-) 13.1**  *(0.003)* | **(-) 8.4**  *(0.028)* | **(-) 7.1**  *(0.039)* | **(-) 8.3**  *(0.021)* | **(-) 7.1**  *(0.036)* | NS | NS | **(-) 11.8**  *(0.005)* | **(-) 12.5**  *(0.007)* | **(-) 10.7**  *(0.010)* | **(-) 9.3**  *(0.044)* |
|  | ν_1_PO_4_/Proline | NS | NS | NS | NS | NS | NS | NS | NS | NS | NS | NS | NS |
|  | ν_1_PO_4_/CH_2_ | **(-) 7.4**  *(0.025)* | **(-) 5.7**  *(0.046)* | NS | NS | **(-) 5.0**  *(0.031)* | **(-) 6.1**  *(0.034)* | NS | NS | **(-) 26.6**  *(<0.001)* | **(-) 24.8**  *(<0.001)* | **(-) 15.1**  *(0.016)* | **(-) 12.9**  *(0.029)* |
| Carbonate  substitution | CO_3_/ν_1_PO_4_ | **(-) 16.0**  *(0.002)* | **(-) 13.8**  *(0.012)* | **(-) 8.7**  *(0.031)* | **(-) 8.3**  *(0.034)* | **(-) 9.1**  *(0.034)* | **(-) 9.2**  *(0.020)* | NS | NS | **(-) 8.1**  *(0.033)* | **(-) 6.9**  *(0.043)* | NS | NS |
| Crystallinity | 1/FWHM  (ν_1_PO_4_) | NS | NS | NS | NS | **(-) 13.3**  *(0.012)* | **(-) 15.3**  *(0.013)* | **(-) 10.1**  *(0.048)* | **(-) 10.7**  *(0.021)* | NS | NS | NS | NS |
| Matrix Maturity | ~I_1670_/I_1690_ | NS | NS | NS | NS | NS | NS | NS | NS | NS | NS | NS | NS |
| Helical Status | ~I_1670_/I_1640_ | **(-) 32.4**  *(<0.001)* | **(-) 28.8**  *(<0.001)* | **(-) 23.7**  *(<0.001)* | **(-) 17.9**  *(<0.001)* | **(-) 17.6**  *(<0.001)* | **(-) 15.2**  *(0.006)* | **(-) 10.4**  *(0.022)* | **(-) 9.8**  *(0.043)* | **(-) 47.4**  *(<0.001)* | **(-) 40.1**  *(<0.001)* | **(-) 27.5**  *(<0.001)* | **(-) 23.6**  *(<0.001)* |
| Helical Status | ~I_1670_/I_1610_ | **(-) 14.1**  *(0.003)* | **(-) 13.8**  *(0.002)* | **(-) 11.7**  *(0.010)* | **(-) 7.6**  *(0.026)* | **(-) 9.6**  *(0.023)* | **(-) 8.8**  *(0.013)* | **(-) 7.1**  *(0.033)* | **(-) 6.5**  *(0.048)* | **(-) 17.1**  *(0.001)* | **(-) 13.0**  *(0.004)* | **(-) 8.9**  *(0.045)* | **(-) 6.6**  *(0.048)* |


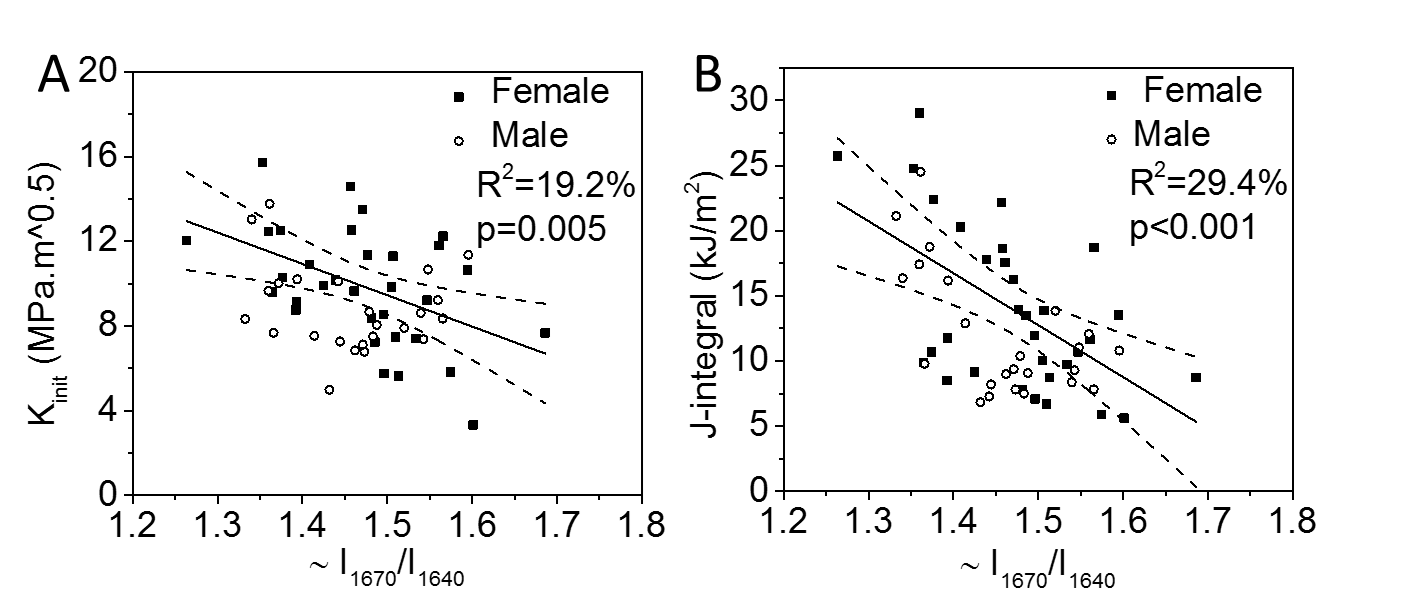


**Supplemental Figure 3:** When collecting only five Raman spectra from one longitudinal surface using fiber optic RS, the correlations between (A) K_init_ and ~I_1670_/I_1640_, and between (B) J-int and ~I_1670_/I_1640_ remained significant with lower R^2^ values compared to the same ratio obtained from ten Raman spectra collecting from both longitudinal surfaces.

**
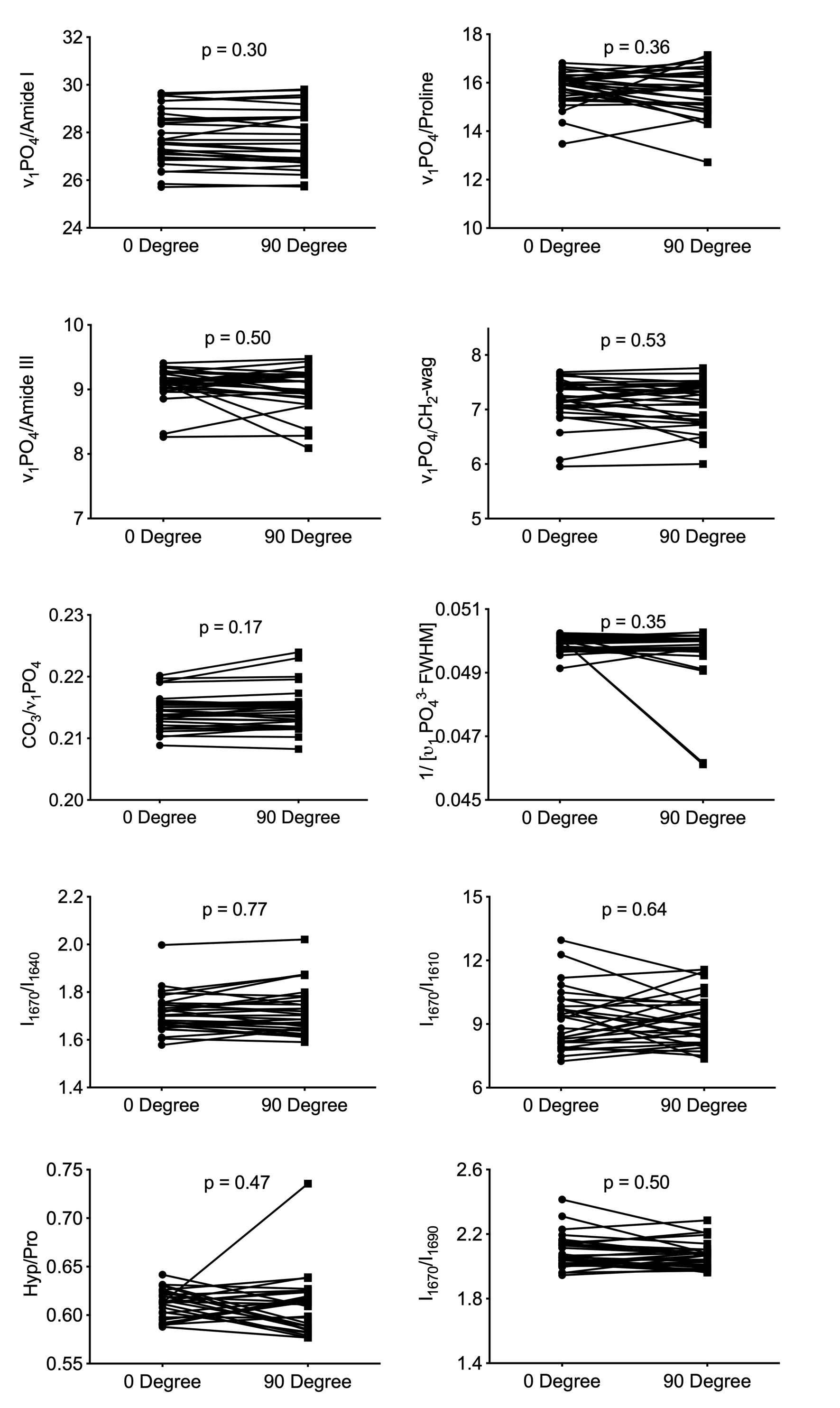
**

**Supplemental Figure 4:** No apparent effect of inherent polarization bias on RS measures. Ten spectra per orientation were acquired from 3 bone specimens (different donors: F/60 yo, M/63 yo, and M/84 yo) using the fiber optic probe-based Raman system before and after rotating the bone sample by 90 degrees. The peak ratios did not depend on orientation, indicating RS measures with fiber optic probe-based Raman system were not significantly affected by inherent polarization bias. P-values are from the Wilcoxon match-pairs signed rank test (GraphPad Prism).

**
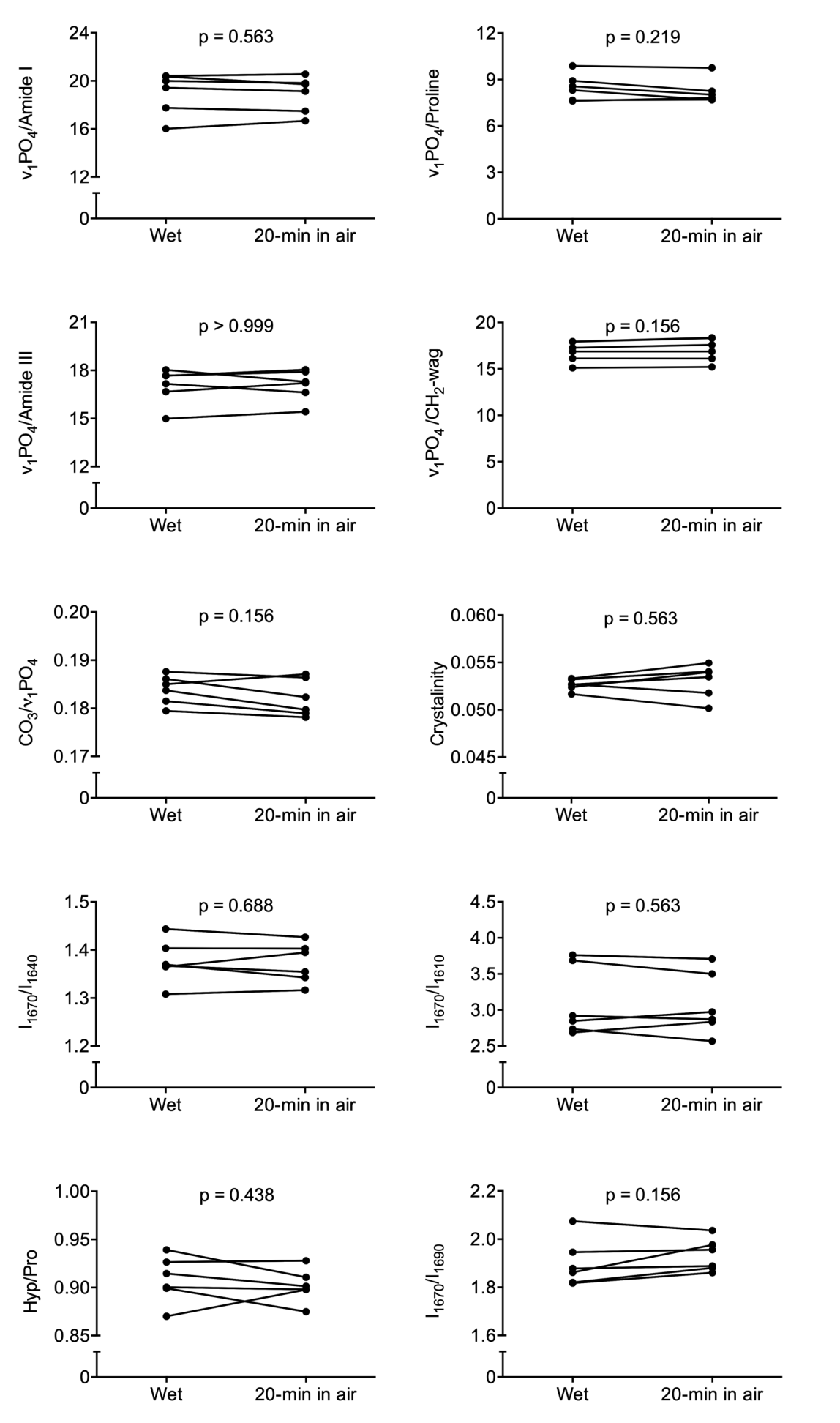
**

**Supplemental Figure 5:** No apparent partial dehydration effect on RS measures. When collecting spectra (average of 10 per time point) from the same area of human cortical bone (6 human donors: F/71 yo, F/70 yo, F/69 yo, F/67 yo, M/97 yo, and M/46 yo,) before (Wet) and after 20-min in air using a research-grade RS system, the calculated RS measures did not differ significantly than control ones, indicating RS measures were not significantly affected over the time to acquire Raman spectra from multiple sites. P-values are from the Wilcoxon match-pairs signed rank test (GraphPad Prism).


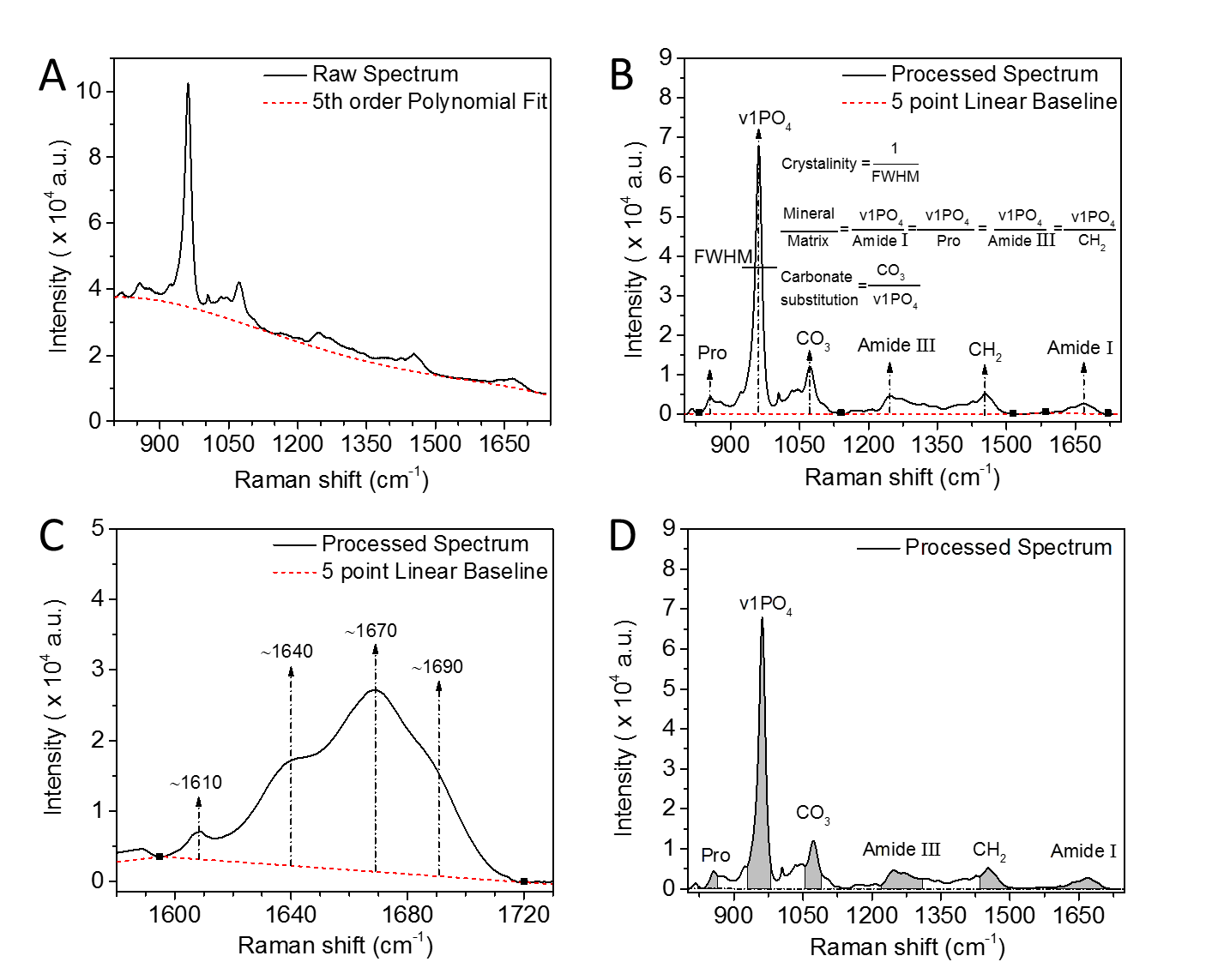


**Supplemental Figure 6:** (A) Background fluorescence was removed from all averaged spectra by subtracting a 5th-order polynomial from the base of the raw spectra (i.e., intensity values were not normalized to the overall mean). (B) The background corrected spectra were further smoothed to improve signal-to-noise ratio using a proprietary de-noising (D-n) algorithm provided by the LabSpec 5 software. Before calculating peak heights, a secondary 5 point linear baseline was applied to account for the possibility of residual fluorescence. (C) The heights of sub-peaks, as identified by the second derivative spectrum, were directly determined from the baseline of the Amide I band. (D) In an alternative determination of the Raman properties, the area of each peak of interest was found by integrating from 835 to 865 cm^-1^ for the Proline peak, from 930 to 980 cm^− 1^ for the ν_1_PO_4_ peak, from 1055 to 1090 cm^− 1^ for the CO_3_ peak, from 1220 to 1310 cm^− 1^ for the Amide III peak, from 1435 to 1500 cm^− 1^ for CH_2_-wag peak, and from 1590 to 1720 cm^− 1^ for the Amide I peak. These peak areas are modified from those used in Hammond et al.[^1^](#_ENREF_1) to match our Raman spectrum.


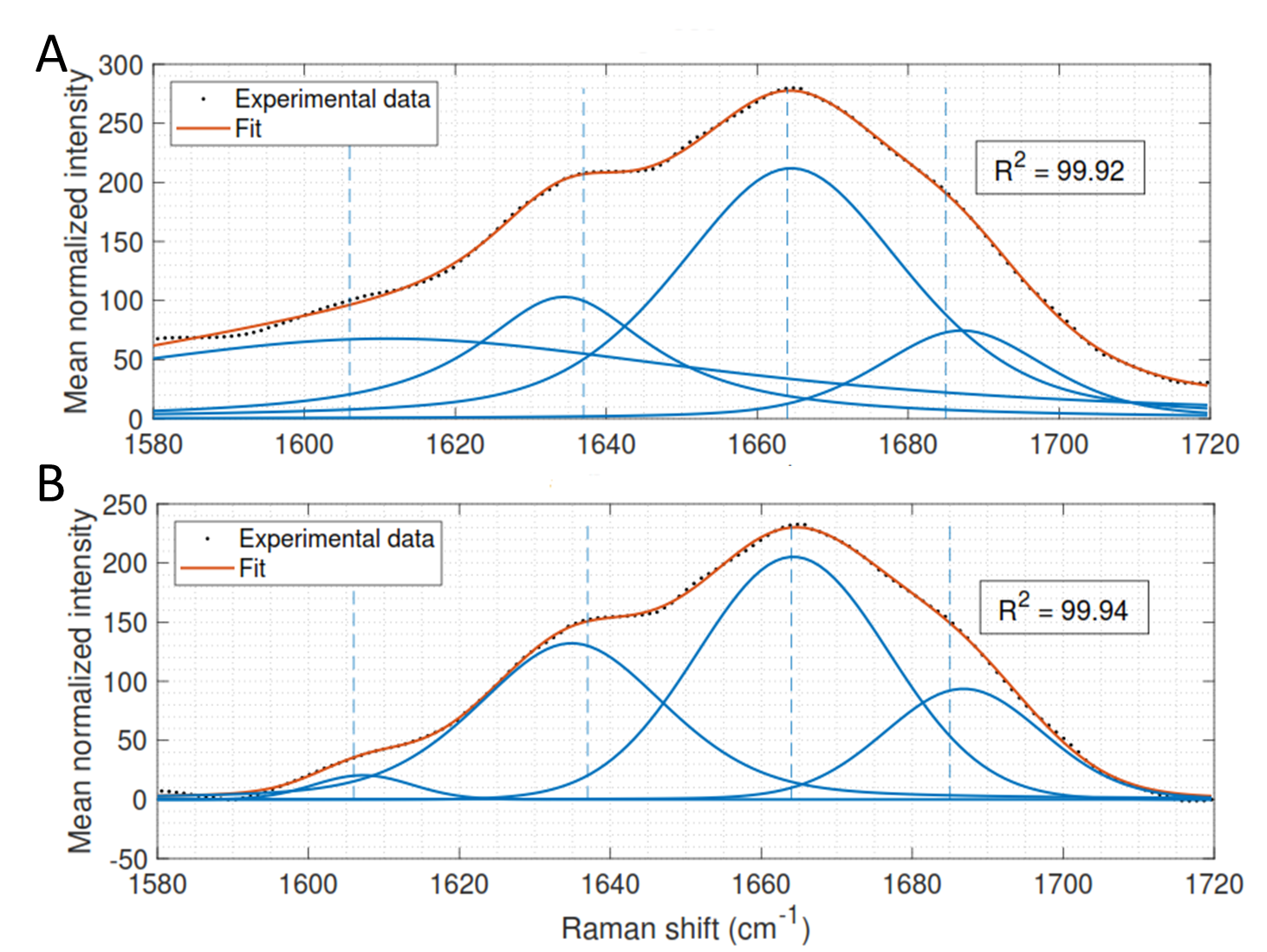


**Supplemental Figure 7:** Amide I sub-band fitting. (A) Without applying a secondary linear baseline correction to the Amide I band, the final sub-bands are irregular. (B) Four sub-bands were consistently fit to the Amide I band after smoothing each spectra and applying a secondary linear baseline correction. The vertical dashed lines indicate the initial position of the 4 sub-bands as identified by the local minima of the second derivative.

**Supplemental Table 3:** P-values obtained from the general linear models for the combinations of age, sex, and their interaction that explain the variance in fracture toughness and RS properties. Interaction was first entered in the model and successively eliminated when not significant (p<0.05) before calculating p-values.

| Research-grade RS | *K_init_* | *K_grow_* | *J-int* | ν_1_PO_4_/Amide I | ν_1_PO_4_/Amide III | ν_1_PO_4_/Proline | ν_1_PO_4_/CH_2_ | CO_3_/ν_1_PO_4_ | 1/FWHM  (ν_1_PO_4_) | I_1670_/I_1610_ | I_1670_/I_1640_ | I_1670_/I_1690_ |
| --- | --- | --- | --- | --- | --- | --- | --- | --- | --- | --- | --- | --- |
| Age | **<0.001** | **0.010** | **0.001** | **0.009** | **0.001** | **0.003** | **0.004** | **<0.001** | **0.001** | **<0.001** | **<0.001** | **0.008** |
| Sex | **0.031^a^** | 0.912 | 0.078 | 0.052 | 0.109 | 0.830 | 0.460 | **0.005** | 0.892 | 0.893 | 0.173 | 0.160 |
| Age x Sex | N/A^b^ | N/A | N/A | **0.004** | **0.047** | N/A | N/A | N/A | N/A | N/A | N/A | N/A |
| Fiber optic RS | *K_init_* | *K_grow_* | *J-int* | ν_1_PO_4_/Amide I | ν_1_PO_4_/Amide III | ν_1_PO_4_/Proline | ν_1_PO_4_/CH_2_ | CO_3_/ν_1_PO_4_ | 1/FWHM  (ν_1_PO_4_) | I_1670_/I_1610_ | I_1670_/I_1640_ | I_1670_/I_1690_ |
| Age | **<0.001** | **0.039** | **0.001** | 0.778 | **0.002** | 0.833 | **0.015** | **<0.001** | 0.122 | **0.002** | **0.003** | **0.024** |
| Sex | **0.047^c^** | 0.767 | 0.124 | 0.683 | 0.144 | 0.460 | 0.135 | 0.128 | 0.872 | 0.866 | 0.568 | 0.101 |
| Age x Sex | N/A | N/A | N/A | N/A | **0.034** | N/A | N/A | N/A | N/A | N/A | N/A | N/A |

^a^The significance of sex as a covariate was due to two donors. Removing either donor (N=57), the p-value was greater than 0.05. Removing both donors (N=56), the p-value was greater than 0.1. ^b^Not applicable because the term was not significant and therefore not included in the best-fit model. See following Supplemental Fig. 2 for regressions. ^c^The significance of sex as a covariate was due to two donors. Removing either donor (N=55) or both (N=54), the p-value was greater than 0.05.

**Reference**

1. Hammond MA, Gallant MA, Burr DB, et al. Nanoscale changes in collagen are reflected in physical and mechanical properties of bone at the microscale in diabetic rats. *Bone*. **60**, 26-32. (2014).
